# Method for the technical, financial, economic and environmental pre-feasibility study of geothermal power plants by RETScreen – Ecuador’s case study

**DOI:** 10.1016/j.mex.2018.05.010

**Published:** 2018-05-21

**Authors:** Diego Moya, Juan Paredes, Prasad Kaparaju

**Affiliations:** a Institute for Applied Sustainability Research (iSUR), Av. Granados E13-55 e Isla Marchena, No.44, Quito, 170503, Ecuador; b Carrera de Ingeniería Mecánica, Facultad de Ingeniería Civil y Mecánica, Universidad Técnica de Ambato, Avd. Los Chasquis y Rio Payamino, 1801314, Ambato, Ecuador; c Department of Chemical Engineering, and the Grantham Institute - Science and Solutions for a Changing Planet DTP, Imperial College London, London, SW7 2AZ, UK; d Griffith School of Engineering, Griffith University, Nathan Campus, 4111 Queensland, Australia

**Keywords:** Modelling of geothermal power plant pre-feasibility studies, Geothermal applications, Techno-economic, Financial, RETScreen

## Abstract

RETScreen presents a proven focused methodology on pre-feasibility studies. Although this tool has been used to carry out a number of pre-feasibility studies of solar, wind, and hydropower projects; that is not the case for geothermal developments. This method paper proposes a systematic methodology to cover all the necessary inputs of the RETScreen-International Geothermal Project Model. As case study, geothermal power plant developments in the Ecuadorian context were analysed by RETScreen-International Geothermal Project Model. Three different scenarios were considered for analyses. Scenario I and II considered incentives of 132.1 USD/MWh for electricity generation and grants of 3 million USD. Scenario III considered the geothermal project with an electricity export price of 49.3 USD/MWh. Scenario III was further divided into IIIA and IIIB case studies. Scenario IIIA considered a 3 million USD grant while Scenario IIIB considered an income of 8.9 USD/MWh for selling heat in direct applications. Modelling results showed that binary power cycle was the most suitable geothermal technology to produce electricity along with aquaculture and greenhouse heating for direct use applications in all scenarios. Financial analyses showed that the debt payment would be 5.36 million USD/year under in Scenario I and III. The correspindig values for Scenario II was 7.06 million USD/year. Net Present Value was positive for all studied scenarios except for Scenario IIIA. Overall, Scenario II was identified as the most feasible project due to positive NPV with short payback period. Scenario IIIB could become financially attractive by selling heat for direct applications. The total initial investment for a 22 MW geothermal power plant was 114.3 million USD (at 2017 costs). Economic analysis showed an annual savings of 24.3 million USD by avoiding fossil fuel electricity generation. More than 184,000 tCO_2_ eq. could be avoided annually.

## Graphical abstract

**Figure:**
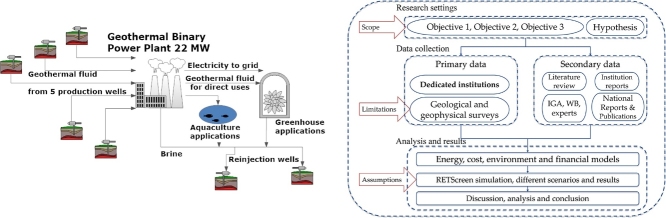

Specifications Table

**Table:** 

Subject area	Energy
More specific subject area	*Geothermal energy – power plant technology and direct uses*
Method name	*Modelling of geothermal power plant pre-feasibility studies*
Name and reference of original method	*RETScreen-International Geothermal Project Model*
Resource availability	*RETScreen*

## Method details

The RETScreen International Clean Energy Project Analysis Software is a feasibility study tool to evaluate energy production, life-cycle costs and greenhouse gas emission reductions for various renewable energy technologies. RETScreen software has been developed by the Ministry of Natural Resources, Canada which offers a proven methodology focused on the pre-feasibility and feasibility studies, rather than developing a custom-developed methodology. In this study, the RETScreen modelling tool was used for the feasibility analysis [1,2]. This model evaluates the energy production of different clean and renewable technologies including life-cycle costs and greenhouse gas emissions (GHG) emission reductions [[1], [2], [3], [4], [5]]. Furthermore, it provides standardised and integrated financial analysis, sensitivity and risk analysis in order to determine the financial viability and risk of the project [3,[5], [6], [7]].

**Fig. 1:**
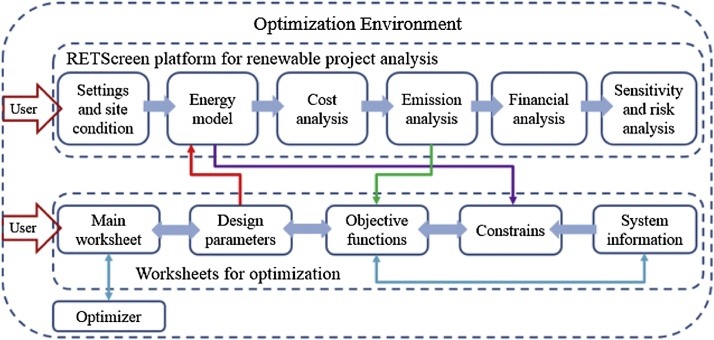
RETScreen model flow chart showing the five-step standard analysis and design parameters [3]. RETScreen modelling includes the Energy Model, the Greenhouse Gas Emission Reduction Analysis Model, the Financial Analysis model (FAM), and the Sensitivity and Risk Analysis Models (SRAM). The FAM includes debt payments, pre-tax and after-tax cash flows, asset depreciation, income tax and financial feasibility indicators, while the SRAM includes the Monte Carlo simulation, impact graph, median and confidence interval, and the risk analysis model validation.

Fig. 1 illustrates the five steps required to complete the analysis: The Energy Model, the Greenhouse Gas Emission Reduction Analysis Model, the Financial Analysis model (FAM), and the Sensitivity and Risk Analysis Models (SRAM) [8]. The FAM includes debt payments, pre-tax and after-tax cash flows, asset depreciation, income tax and financial feasibility indicators, while the SRAM includes the Monte Carlo simulation, impact graph, median and confidence interval, and the risk analysis model validation [2]. Data was collected from reports published by CELEC, INER-MEER, IGA (International Geothermal Association), WB (World Bank) and PUGR-E (Plan for the Utilization of Geothermal Resources in Ecuador – unpublished government document).

### Statement of assumptions

For financial analyses, some assumptions were established based on the literature data [[9], [10], [11]]. This study found that the costs have increased by approximately 50% during the period 2009–2016. Therefore, the assumptions for the total investment costs were based on this growth rate. The following sections present the procedure for technical, financial and economic data analyses.

#### Technical data

In RETScreen model, technical analysis was defined by the energy model. In the technical analysis, electricity energy matrix, and how geothermal power generation and direct use technologies would be relevant to the goal of changing the Ecuadorian energy and productivity matrices were evaluated. Based on the recent and previous studies published by CELEC-EP, the potential geothermal prospects for electricity generation and direct applications have been conceptualised with their technical feasibility to be developed [[12], [13], [14]]. Finally, the sub-problems related to the state of the electricity sector framework of Ecuador to develop geothermal projects were also considered in the analysis.

Table 1 presents the data required to proceed with the technical analysis using RETScreen. Based on the literature, parasitic load of 10% and transmission losses of 2% were assumed in this study [11,15]. Consequently, the effective full load power capacity for a 22 MW geothermal power plant was estimated at 19.36 MW.

**Table 1:** Data used to calculate the Power capacity and Grid exported electricity of the proposed configuration for the Energy Model by RETScreen software.

Technical item	Quantity	Unit	Source
Installed capacity of geothermal power	Up to 81 81,000	MW kW	[16]
Availability	97	%	[15]
Production wells	5	wells	[17,18]
Reinjection wells	2	wells	[17,18]
Total wells	7	wells	
Steam flow 5 production wells	65 234,000 1,170,000	kg/s kg/h kg/h	[18,19]
Temperature (fluid in reservoir)	210 - 350	°C	[16]
Operation pressure	6 600	bar kPa	[19,20]
Steam temperature	200	°C	[16]
Back pressure	3.95 395	bar kPa	[20]
Steam turbine efficiency	80	%	[21]
Minimum capacity	50	%	[2]
Electricity export rate	132.1	USD/MWh	[22]
Parasitic loads	10 2.2	% MW	[11]
Transmission loss	2 0.44	% MW	[11]
Construction time	18	months	[11]
Life time	15 25	Years Years	[23] [16]

#### Financial data

In the financial analysis, detailed investment costs were assessed to formulate the most complete financial model for the development of geothermal energy projects. This approach will identify the limitations and opportunities for investment in geothermal power projects in the Ecuadorian context. The role of funding bodies, both public and private, current carbon and energy tax policies, and other related frameworks were also considered in this analysis. The input data required by the RETScreen Cost Analysis Model are presented in detail in Table 2.

**Table 2:** Input data on investment and operating costs for the geothermal power plant in Ecuador used based on literature data and adapted to the Ecuadorian context as of December 2016 [10,11,24].

Capital costs – Initial investment		
Exploration	4,500,000	USD
Well field development (7 wells: 5-production, 2-injection)	35,000,000	USD
Plant equipment (using 1.5 scale factor)	57,000,000	USD
Permits for land use	750,000	USD
Interconnection	375,000	USD
Overheads profit	11,715,000	USD
Construction schedule	18	Months
Interest during construction	6	%
Contingencies	6.5	%
Total	109,340,000	USD

Operating costs – Annual costs		
**LABOR**		
Plant manager – SP10 (1x$2308x12months)	27,696	USD
Plant operators – SP3 (8x$986x12 months)	94,656	USD
Mechanic – SP3 (1x$986x12months)	11832	USD
Other labour – SP1 (1x$817x12months)	9804	USD
Total	143,988	USD

**PLANT**		
Turbine/generator	37,500	USD
Electric and control systems	64,500	USD
Cooling systems	9,000	USD
Auxiliary systems	19,500	USD
Cooling water and chemicals	70,500	USD
Miscellaneous and consumables^a^	75,000	USD
Total	201,000	USD

**WELL FIELD**		
Well clean	138,000	USD
Brine chemicals	75,000	USD
Miscellaneous^a^	52,500	USD
Total	213,000	USD

Major overhaul (maintenance), fees, resource costs		
**MAJOR OVERHAUL**		
Plant (L+M), every 3 years	1,161,000 387,000	USD/3 yrs. USD/yr
Labour – L (90$/h, 400h)	36,000	USD/3 yrs.
Materials, parts - M	1,125,000	USD/3 yrs.
Well maintenance (every 2 years)	75,000 37,500	USD/2 yrs USD/yr.
Well replacement (every 5 years)	3,450,000 690,000	USD/5 yrs. USD/yr

**FUEL RESOURCE**		
Community benefits (3% of total electricity sales)	229,979	USD
Reservoir management	37,500	USD
Make-up water	9,750	USD
Land lease fees	8,400	USD
Total	**1,400,129**	**USD**

SP10, SP3, SP1 are the classification levels of public servants in the Ecuadorian system.

a These values are included in the % of contingencies.

In annual costs, the operation and maintenance of the power plant was calculated based on three individual costs: parts and labour, well field and contingencies. The parts cost was related to the parts required for the turbine-generator, the electric and control systems, the cooling system, auxiliary systems, and cooling water and chemicals. The annual labour costs were calculated at an operating labour of 11 staff: 1 plant manager, 8 plant operators, 1 mechanic and 1 labourer. These costs have been taken from the unified scale of monthly salaries from the Ministry of Labour Relations of Ecuador [24]. RETScreen financial analysis model calculates two main financial indicators: debt payment and Net Present Value (NPV). Debt payment is the sum principal portion increases with time, and the interest portion decreases with time. On the other hand, NPV is the value of all future cash flows in today’s currency discounted at the proposed discounted rate [2]. A positive NPV indicates that the project is feasible in financial terms. Finally, the second set of financial indicators i.e. simple and equity payback periods are analysed [25]. Simple payback represents the length of time for the proposers to recoup their initial investment, while equity payback represents the length of time for the owner to recoup its own initial investment [2].

#### Economic data

RETScreen Cost Analysis Model includes costs related to development, engineering, power system and balance of systems and miscellaneous, for initial costs; and operation and maintenance, for annual costs [3]. In the economic analysis, specific micro-economic and macro-economic variables were considered [26]. From the micro-economic point of view, the electricity market structure of Ecuador was studied in order to determine if there were any structures to support geothermal developments. In addition, the demand and supply of renewable energy and how geothermal energy could play an important role in the diversification of the Ecuadorian Energy Matrix was analysed. From the macro-economic point of view, four variables were addressed: the share of renewable energy production; and finally, employment opportunities that geothermal projects may establish was also studied.

#### Greenhouse gas emissions data

In the GHG analysis, a comprehensive Ecuador’s energy matrix was considered by including primary energy and electricity consumptions. RETScreen Greenhouse Gas Emissions Analysis Model provides the carbon dioxide (CO_2_,), methane (CH_4_), and nitrous oxide (N_2_O) emissions that can be avoided on replacing fossil fuel with renewable energy resource [2,8]. Table 3 presents the input data on the share of each fuel type in the country’s fuel mix, electricity generation efficiency, and the transmission and distribution (T&D) losses [[27], [28], [29]] along with GHG emission factors used for calculating GHG emissions.

**Table 3:** Input data for calculating the greenhouse gas emissions in the base case electricity scenario [2,27,29,30,31,32].

Fuel type	Fuel mix	CO2 emission factor	CH4 emission factor	N2O emission factor	Electricity generation efficiency	T&D losses	GHG emission factor
	%	kg/GJ	kg/GJ	kg/GJ	%	%	tCO2/MWh
Oil	90.0	74.12	0.0029	0.0019	28.60	12.4	1.075
Natural gas	4.0	49.36	0.0036	0.0009	40.80	12.4	0.501
Hydro	4.0	0.00	0.0000	0.0000	100.00	12.4	0.000
Biomass	2.0	0.00	0.0299	0.0037	23.30	12.4	0.033
Electricity mix	100.0	272.05	0.0136	0.0073		12.4	0.988

#### Scenarios

Three likely scenarios were studied. Scenario I was based on a project life of 25 years, which is the usual term for World Bank geothermal projects [33]. Scenario II was based on a project life of 15 years, which is the usual term for the National Electricity Council, CONELEC, renewable energy projects [23]. For Scenario I and II, an incentive and grants of 3 million USD were considered, an amount already provided by the government. Scenario III does not take into account the government incentive of 132.1 USD/MWh and the project was considered as fossil-fuel power plant project at 49 USD/MWh [23]. Within Scenario III, two separate cases were considered based on the availability of different financial incentives viz., other grants, direct application, GHG reduction income and Clean Energy production income. In Scenario IIIA, electricity export price at 49.3 USD/MWh and 3 million USD grant was considered. On the other hand, grants, incentives and direct applications of heat were considered in Scenario IIIB. In addition, Scenario IIIB also assumes 20 million USD government grants and an income of 8.9 USD/MWh for the sale of heat for direct applications estimated at 115 MW h per year. Finally, two Clean Development Mechanisms (CDM) of funding were proposed. For GHG reduction income, 7 USD/tCO_2_ avoided was assumed [34]. Similary, 0.01 USD/kWh of clean energy produced was assumed under Clean Energy production income [1], which is assumed as a likely incentive if a Geothermal Law comes into existence in the country.

### Scope and limitations

This study does not engage with geology, geophysics and exploration studies of geothermal resources. However, CELEC-EP has provided evidence of cited studies, which support the selection of potential geothermal prospects to harvest high and low enthalpy energy for electricity generation and to use in direct applications. The specific document on which this study was based is the PUGR-E, elaborated by the MEER and provided by CELEC-EP for this study [12]. It is beyond the scope of this study to conduct laboratory experiments to support the technical analysis. The technical analysis of the penetration of geothermal energy systems in the energy and productivity matrix of Ecuador was based on a detailed and systematic review of the related scientific and academic literature of the technologies currently in use to harvest energy from geothermal resources. The study would suggest plant configurations to produce electricity and thermodynamic cycle configurations for direct use of geothermal resources. The boundaries of the financial analysis were subjected to the current financial framework of Ecuador. However, it was proposed to conduct this analysis using three scenarios. The first scenario was based on the current financial environment, which is public funding. The second was a mixed funding between public and private funds. While the third scenario was studied without any incentives but taking into account other funding sources.

A full discussion of micro- and macro-economic variables lies beyond the scope of this study. Therefore, the study focused on the economic aspects previously described. Nevertheless, the analysed economic variables should be more than adequate to predict the economic impact of the penetration of geothermal energy projects in the Ecuadorian economy. In the case of replicability, the authors suggest to update data and convert it to the context in consideration. An application of this methodology is fully described in [35].
